# Specific Neural Coding of Complex Neural Network Based on Time Coding Under Various Exterior Stimuli

**DOI:** 10.3390/biomimetics10030162

**Published:** 2025-03-06

**Authors:** Lei Guo, Zhixian Wang, Yihua Song, Huan Liu

**Affiliations:** 1 Tianjin Key Laboratory of Bioelectromagnetic Technology and Intelligent Health, School of Health Sciences and Biomedical Engineering, Hebei University of Technology, Tianjin 300131, China; 202232902029@stu.hebut.edu.cn (Z.W.); 202212901005@stu.hebut.edu.cn (Y.S.); 202011402002@stu.hebut.edu.cn (H.L.); 2 State Key Laboratory of Reliability and Intelligence of Electrical Equipment, Hebei University of Technology, Tianjin 300131, China

**Keywords:** spiking neural network, complex network, time coding, specific neural coding, synaptic plasticity

## Abstract

Specific neural coding (SNC) forms the basis of information processing in bio-brain, which generates distinct patterns of neural coding in response to corresponding exterior forms of stimulus. The performance of SNC is extremely dependent on brain-inspired models. However, the bio-rationality of a brain-inspired model remains inadequate. The purpose of this paper is to investigate a more bio-rational brain-inspired model and the SNC of this brain-inspired model. In this study, we construct a complex spiking neural network (CSNN) in which its topology has the small-word property and the scale-free property. Then, we investigated the SNC of CSNN under various strengths of various stimuli and discussed its mechanism. Our results indicate that (1) CSNN has similar neural time coding under same kind of stimulus; (2) CSNN has significant SNC based on time coding under various exterior stimuli; (3) our discussion implies that the inherent factor of SNC is synaptic plasticity.

## 1. Introduction

Bio-brain exhibits remarkable information processing ability [1]. Neural information coding is crucial for facilitating this processing. Various responses are formed in response to various stimuli, which is essential for the brain to differentiate between various stimuli to achieve advanced brain functions [2,3]. The performance of SNC is extremely dependent on brain-inspired models. However, the bio-rationality of a brain-inspired model remains inadequate as they do not fully capture the structure and function of real neural systems. This research gap limits the ability of current models to replicate the complexities of biological neural coding mechanisms. Spiking neural network (SNN), inspired by the brain [4,5], can drive advancements in artificial intelligence through research on their specific neural coding. An SNN architecture consists of a neuron model, a synaptic plasticity model, and a network topology.

Bio-neurons act as units for processing neural information within the brain, while neuron models are mathematical representations that replicate electrophysiological characteristics of bio-neurons. Many types of neuron models have been developed by researchers, including the Hodgkin–Huxley (H-H) neuron model, the leaky integrate-and-fire (LIF) neuron model, the Izhikevich neuron model, and so on. Since the H-H neuron model incurs significant computational expense [6], the LIF neuron model reduces computational expense but fails to accurately replicate the firing behavior observed in bio-neurons [7]. The Izhikevich neuron model can reflect the firing activity of bio-neurons well while maintaining a reduced computing expense [8]. Therefore, it is extensively utilized in the construction of SNNs [9,10].

Bio-synapses serve as the fundamental components for transmitting neural information among bio-neurons [11]. Excitatory synapses (ESs) have the capacity to enhance the effectiveness of neural information transfer [12], whereas inhibitory synapses (ISs) have the capacity to decrease the speed and sensitivity of neural information transfer [13]. Biological studies have shown that ISs, in conjunction with ESs, play a crucial role in the regulation of neural activity in the bio-brain. For instance, Xue et al. [14] found that the proportion of ISs rose alongside heightened neuronal excitability by measuring how neurons of young mice responded to optical stimuli, which reflects excitation–inhibition balance in anatomy. Inspired by bio-synapses, synaptic plasticity models co-regulated by ESs and ISs are utilized to construct SNNs. Zhao et al. [15] constructed an SNN with both ES and IS models. They discovered that this SNN outperforms an SNN without an inhibitory synapse model on MNIST datasets. For bio-synapses, the neurotransmitter dispersion induces a synaptic time delay (STD), which is stochastically distributed within the interval [0.1, 40]  ms [16]. Hence, synaptic plasticity models with STD have been introduced in SNNs. Zhang et al. [17] developed an SNN with STD, which demonstrated the ability to accurately reproduce the expected spike sequence and achieved better accuracy than an SNN without an STD on the TIDIGITS speech recognition task. However, in this study, the STD was set to a fixed value that did not conform to the bio-STD interval [0.1, 40]  ms. Hence, on the basis of co-regulation of ESs and ISs, a synaptic plasticity model with STD conforming to the bio-STD can improve the performance of SNNs.

The topology decides the forms of connection between neurons in a brain network. According to principles of complex network theory, networks are classified according to their topological structure into regular, random, and complex networks. Among these, complex networks encompass small-world (SW) networks and scale-free (SF) networks [18]. The SW networks exhibit both a high mean clustering coefficient (CC) and a low mean shortest path length (SPL) [19]. The SF network exhibits the degree distribution of nodes adheres to a power-law distribution, characterized by significant heterogeneity, which endows it with robust fault tolerance [20]. Biological research has shown that bio-functional brain networks (FBN) are complex networks with SW and/or SF properties. Van et al. [21] generated FBNs of 28 healthy subjects, finding that these networks exhibited SW and SF properties. Liu et al. [22] examined the SW property of FBNs in 31 schizophrenia patients and 31 healthy individuals, revealing that SW property was absent in schizophrenia patients. Stylianou et al. [23] generated FBNs for 15 Parkinson’s disease patients to investigate the effect of treatment of dopaminergic on SF property and found that SF property was normal by the treatment. Based on results for FBNs, researchers have constructed SNNs with the topology of SW and/or SF properties. Tsakalos et al. [24] developed a small-world spiking neural network (SWSNN) by utilizing Watts and Strogatz (WS) algorithm for topology generation. They discovered that this SWSNN achieved superior recognition accuracy compared to a two-layered SNN on the image dataset. Reis et al. [25] constructed an SFSNN utilizing Barabási–Albert (BA) algorithm and investigated synchronization. They discovered that the SFSNN could inhibit burst synchronization under the exterior disturbance of light pulses while still maintaining low synchronization after the disturbance had stopped for an extended period. In a previous study [26], we constructed SWSNN and SFSNN to explore anti-interference capacity under pulse noise. Our findings revealed that SWSNN was more robust than SFSNN, which indicates that anti-interference capacity of these two SNNs is affected by topology. Based on the topological characteristics of bio-FBN, the complex spiking neural network (CSNN) exhibiting SW property and SF property can enhance the bio-rationality of brain-inspired models.

The bio-brain responds to exterior stimuli through neural coding [27]. Callier et al. [28] investigated the reaction of populations of neurons in monkey’s brain when exposed to skin pressure marks. Their study revealed that these neurons can encode the timing, location, and magnitude of skin indentation quickly and consistently, indicating that population coding can effectively encode features of contact events. Inspired by these biological findings, researchers have examined the coding of brain-inspired models. Zhu et al. [29] examined energy consumption of an SNN. They discovered that the distribution of network energy is positively associated with the strength of coupling between neurons, implying that energy coding is a useful tool for assessing cost-effectiveness of the brain-inspired model. Du et al. [30] studied the firing activity of an SNN under a sinusoidal induced electric field (IEF) using the inter-spike intervals (ISI). They found that the ISI gradually became an integer multiple of the firing period over time, forming a stable neural coding for IEF with random phase noise. Biological results have demonstrated that the bio-brain can form specific patterns of neural coding in response to various forms of exterior stimulus. For example, Stephanie et al. [31] observed neural activity in the brains of mice in response to different forms of taste stimulus and found that there was obvious specificity in the population coding in the gustatory cortex, indicating that different taste stimuli allow mice to produce different coding patterns. Therefore, the SNC can generate distinctive coding patterns that correspond to specific exterior stimuli. The performance of SNC is extremely dependent on brain-inspired models. However, the bio-rationality of the topology in current brain-inspired models remains deficient. This gap hinders the potential of neural coding in applications.

In order to deal with the above challenges, we propose a brain-inspired model with bio-rationality and investigate the SNC of CSNN under various strengths of various stimuli. In this study, we construct a CSNN with a topology that exhibits SW and SF properties, where the nodes are represented by Izhikevich neuron models, and the edges consist of synaptic plasticity models incorporating bio-STD, co-regulated by ESs and ISs. This design enhances the bio-rationality of the model, making it more aligned with the bio-brain and its information-processing capabilities. To investigate the SNC of CSNN under various strengths of different stimuli, K-means clustering algorithm and cosine similarity algorithms are employed. K-means clustering algorithm is well-suited for this task, efficiently grouping coding patterns into clusters and enabling the analysis of coding differences across stimuli, which helps to identify different responses across varying stimuli and allows for a clear analysis of coding differences. Meanwhile, the cosine similarity algorithm quantifies the similarity between coding patterns, and thus enabling a deeper understanding of the similarity within the classes. Since neural coding primarily relies on time coding patterns rather than absolute spike counts, cosine similarity provides an effective measure for evaluating the consistency of SNC under different stimulus. These two strategies complement each other and offer a way to analyze the time neural coding in CSNNs. Furthermore, we discuss the mechanism of the SNC by analyzing the correlation between the SNC and the synaptic plasticity.

The main contributions of this paper are as follows:

- To enhance the bio-rationality of brain-inspired models, a CSNN is proposed with a topology that incorporates both SW and SF properties. The nodes are Izhikevich neuron models, and the edges are represented by synaptic plasticity models with a bio-STD co-regulated by ESs and ISs.
- To investigate the SNC of CSNN under various strengths of various stimuli, the K-means clustering algorithm and cosine similarity algorithm are used. Our results indicate that CSNN exhibits marked time coding similarity under various strengths of same stimulus; the CSNN exhibits marked SNC under various stimuli.
- To elucidate mechanism of the SNC based on CSNN, we conduct a discussion between synaptic weight and SNC, which indicates that the inherent factor of the SNC is synaptic plasticity.

**Figure 1:**
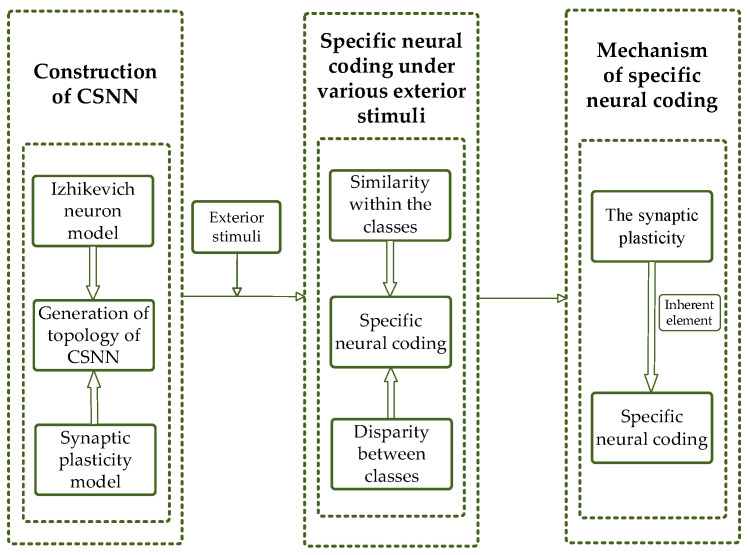
Flowchart of our study.

The following sections are organized as follows: The method of constructing the CSNN is described in Section 2. The SNC of CSNN under various stimuli is proposed in Section 3. Mechanism of the SNC is discussed in Section 4. Finally, a conclusion is presented in Section 5. Our flowchart is shown in Figure 1.

## 2. Materials and Methods

In this section, we describe construction of CSNN and a neural coding approach of CSNN.

### 2.1. Construction of CSNN

To increase the bio-rationality of brain-inspired models, the CSNN is constructed. Then, we investigate the topology characteristics including CC and SPL.

#### 2.1.1. Izhikevich Neuron Model

Based on the description in Section 1, Izhikevich neuron model has a low computational expense and precisely reflects firing activity of bio-neurons, compared with H-H neuron model [6] and LIF neuron model [7]. Thus, we use the Izhikevich neuron model to represent the nodes in CSNN, which is expressed as follows [32]:

(1) $$\left\{\begin{array}{c} dv/dt=0.04v^{2}+5v+140-u+I_{ex}+I_{g}\\ du/dt=a\left(bv-u\right)\\ if\;v\geq 30,\;then\left\{\begin{array}{l} v=c\\ u=u+d \end{array}\right. \end{array}\right.$$

where v represents membrane voltage; u represents recovery variable for v; $I_{ex}$ represents the exterior current; $I_{g}$ represents synaptic current; and a, b, c, and d are dimensionless variables which simulate both excitatory and inhibitory neurons.

#### 2.1.2. Synaptic Plasticity Model

A synaptic plasticity model with bio-STD and co-regulation of ES and IS can enhance the performance of SNNs. Thus, we use this synaptic plasticity model.

An STD is introduced to represent the diffusion of neurotransmitters in bio-synapses, defined as follows [33]:

(2) $$I_{g}\left(t\right)=g\left(t\right)r\left(t\right)\left(E-V_{post}(t)\right)$$

where $I_{g}$ denotes the synaptic current; $V_{post}$ denotes postsynaptic membrane potential; g denotes synaptic weight; and r reflects changes in concentration of the neurotransmitter H, which can be described as follows:

(3) $$\left\{\begin{array}{l} dr/dt=\alpha H\left(1-r\right)-\beta r\\ H={\left[1+\exp(-V_{pre}(t-t_{d}))\right]}^{-1} \end{array}\right.$$

where $V_{pre}$ denotes presynaptic membrane potential; ES weight $g_{ex}$ and IS weight $g_{in}$ are regulated by following rules [34]; α and β denote the constants for the positive and negative combination speeds of the neurotransmitter, respectively; $t_{d}$ is STD which conforms to the bio-STD.

When a postsynaptic neuron j does not detect an action potential from a presynaptic neuron i, $g_{ex}$ and $g_{in}$ slump as follows:

(4) $$\left\{\begin{array}{c} \mu_{ex}(dg_{ex}/dt)=-g_{ex}\\ \mu_{in}(dg_{in}/dt)=-g_{in} \end{array}\right.$$

where $\mu_{ex}$ and $\mu_{in}$ represent the decay constants of the excitatory synaptic conductance and inhibitory synaptic conductance, respectively.

When j detects an action potential from i, $g_{ex}$ and $g_{in}$ change as follows:

(5) $$\left\{\begin{array}{l} g_{ex}\left(t\right)=g_{ex}\left(t\right)+{\bar{g}}_{ex}\\ {\bar{g}}_{ex}=w\left(\Delta t\right)*g_{max} \end{array}\right.$$

(6) $$\left\{\begin{array}{l} g_{\mathrm{in}}\left(t\right)=g_{in}\left(t\right)+{\bar{g}}_{in}\\ {\bar{g}}_{in}=m\left(\Delta t\right)*g_{max} \end{array}\right.$$

If g exceeds $g_{max}$, it is set to $g_{max}$; however, whereas if g<0, it is set to 0. The ES increment ${\bar{g}}_{ex}$ and IS weight increment ${\bar{g}}_{in}$ are regulated by $w\left(∆t\right)$ and $m\left(∆t\right)$, which are the modification functions for the STDP and can be defined as follows:

(7) $$w\left(∆t\right)=\left\{\begin{array}{l} A_{+}\exp(\Delta t/t_{+}),\Delta t<0\\ -A_{-}\exp(-\Delta t/t_{-}),\Delta t\geq 0 \end{array}\right.$$

(8) $$m\left(∆t\right)=\left\{\begin{array}{l} -B_{+}\exp(\Delta t/t_{+}),\Delta t<0\\ B_{-}\exp(-\Delta t/t_{-}),\Delta t\geq 0 \end{array}\right.$$

where Δt denotes the firing interval between the presynaptic and postsynaptic spikes.

#### 2.1.3. Complex Network Topology

The complex network topology exhibiting SW and SF properties can enhance the bio-rationality of brain-inspired models. Thus, we generate complex network topology. The Barrat–Barthelemy–Vespignani (BBV) algorithm is capable of simulating the dynamic evolution in the local edge weights caused by adding new nodes and regulating the mean CC within a large scope [35]. It can construct complex networks of various topologies with the SW and SF properties by adjusting the reconnection probability parameter $P_{n}$. In this study, the BBV algorithm is utilized to generate a complex network topology; selecting an appropriate value for $P_{n}$ is essential to achieve a network with bio-rationality through analysis of its SW property σ [36] and SF property γ  [37].

To obtain an appropriate $P_{n}$ in order to generate a complex network with the SW and SF properties, we generate complex networks within the $P_{n}$ range [0.1, 1] in steps of 0.1. We set network size N is 500 nodes. SW and SF properties of the complex networks for various $P_{n}$ are presented in Table 1.

From these simulations, we observed that when $P_{n}=0.3$, the corresponding γ is 2.15, which closely aligns with the SF property of the human FBN, which is 2 [38]. Similarly, at $P_{n}=0.3$, σ=1.89, which falls within the range of the SW property of the human FBN [39]. Thus, we select $P_{n}=0.3$ to generate a bio-rational network topology to construct the CSNN.

#### 2.1.4. Topological Characteristics of CSNN

In order to evaluate CSNN, we investigated its topological characteristics including CC and SPL.

1. CC

Mean CC denotes the tightness of nodes in a network, thereby illustrating the effectiveness of local information transfer within an SNN [40]. Since the edges of the SNN are weighted, it is necessary to use the mean weighted CC (${\tilde{C}}_{w}$) with the following expression:

(9) $${\tilde{C}}_{w}=\frac{1}{N}\sum_{i=1}^{N}\left(\frac{1}{s_{i}\left(k_{i}-1\right)}\sum_{j,k}\frac{\left(g_{ij}+g_{ik}\right)}{2}a_{ij}a_{jk}a_{ki}\right)$$

where $s_{i}$ denotes the node strength; $g_{ij}$ and $g_{ik}$ denote the synaptic weights; $k_{i}$ denotes the node degree; and $a_{ij}$, $a_{jk}$, and $a_{ki}$ denote adjacent matrices.

2. SPL

The mean SPL denotes the mean of the shortest distance between all pairs of nodes in a network, thereby illustrating the effectiveness of global information transfer within an SNN [40]. Since the edges of the SNN are weighted, it is necessary to use the mean weighted SPL (${\tilde{L}}_{w}$) with the following expression:

(10) $${\tilde{L}}_{w}=\frac{1}{N\left(N-1\right)}\begin{array}{c} min\\ \Upsilon \left(i,j\right)\in \Gamma \left(i,j\right) \end{array}\left[\sum_{m,n\in \Upsilon \left(i,j\right)}\frac{1}{g_{mn}}\right]$$

where $g_{mn}$ denotes synaptic weights; $\Upsilon \left(i,j\right)$ denotes connection between i and  j; and $\Gamma \left(i,j\right)$ denotes the possible pathway from i to j.

### 2.2. The Time Coding of CSNN Under Exterior Stimuli

To evaluate the effectiveness of the brain-inspired model, we investigated the SNC of the CSNN based on time coding under exterior stimuli.

#### 2.2.1. Exterior Stimuli

To investigate SNC of the CSNN under exterior stimuli, three stimuli were employed including white Gaussian stimulus, impulse stimulus, and AC magnetic field stimulus.

1. White Gaussian stimulus

A Gaussian distribution governs the instantaneous intensity $A_{g}$ of white Gaussian noise, and it is expressed as follows:

(11) $$f\left(A_{g}\right)=\frac{1}{\sqrt{2\pi }\sigma_{g}}exp\left(-\frac{{\left(A_{g}-\mu_{g}\right)}^{2}}{2{\sigma_{g}}^{2}}\right)$$

where $\sigma_{g}$ denotes the standard deviation in $A_{g}$; and $\mu_{g}$ is the mean value of $A_{g}$. In this study, the instantaneous intensity $A_{g}$ is continuously added to $I_{ex}$ throughout the 1000 ms.

2. Impulse stimulus

The short duration, huge amplitude, and burst patterns of irregular discontinuous impulse spikes make up impulse noise. The mathematical description is as follows:

(12) $$s(t)=\left\{\begin{array}{l} A_{s},t\in [T_{0},T_{0}+T]\\ 0,else \end{array}\right.$$

where $T_{0}$ denotes the onset time of stimulus; T stands for the duration of the stimulus; and $A_{s}$ stands for the impulse intensity. In this study, $T_{0}$ remains consistent across all neurons, indicating that the impulse stimulus is introduced to each neuron model concurrently at the same initial moment, T is 200 ms. All neuron models experience impulse noise, which is viewed as a current disturbance on the exterior input current $I_{ex}$ in Equation (1).

3. AC magnetic field stimulus

The alternating magnetic field stimulus $V\left(t\right)$ is described as follows:

(13) $$∆V\left(t\right)=A_{c}cos\left(2\pi ft\right)$$

where $A_{c}$ stands for the amplitude of AC magnetic field stimulus; and f is the magnetic field frequency. All of the neuron models are subjected to AC magnetic field stimulus, which is denoted in Equation (1) as a voltage disturbance on the membrane potential v.

#### 2.2.2. Time Coding Method of CSNN

Time coding can be used to encode neural information by capturing the precise moments of the neuronal action potential. One kind of time coding is ISI coding which is based on the disparity between adjacent firing times, which can accurately reflect dynamic neural information [30]. When a neuron model fires an action potential at time t, p-th element of ISI series is defined as follows:

(14) $${ISI}_{p}=t_{p}-t_{p-1}$$

The coding of ISI can be analyzed through three aspects: ISI time domain diagram, ISI histogram, and joint ISI distribution. ISI time domain diagram denotes dynamic variations of ISI of all neurons over time. ISI histogram denotes the rate distribution of ISI within the entire set of ISIs. Joint ISI distribution denotes disparities of neighboring ISIs among all neurons.

1. Coding pattern of CSNN white Gaussian stimulus

**Figure 2:**
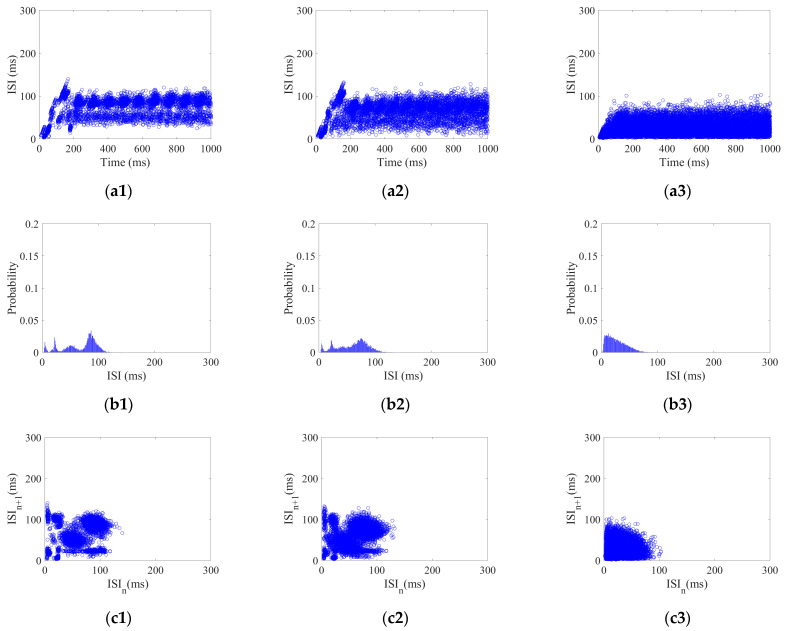
ISI coding pattern of the CSNN under white Gaussian stimulus: (**a1**–**a3**) ISI time domain diagrams; (**b1**–**b3**) ISI histograms; (**c1**–**c3**) joint ISI distributions.

Each neuron in the CSNN received white Gaussian stimulus with a strength range of 5–25 dbW with 2.5 dbW/step. Using white Gaussian stimulus $A_{g}$ of 5, 15, and 25 dbW as examples, their three aspects of ISI coding are shown in Figure 2.

Figure 2(a1–a3) show ISI time domain diagrams under $A_{g}$ of 5, 15, and 25 dbW; Figure 2(b1–b3) show ISI histograms under $A_{g}$ of 5, 15, and 25 dbW; Figure 2(c1–c3) show joint ISI distributions under $A_{g}$ of 5, 15, and 25 dbW. The neural coding pattern illustrated in Figure 2 reflects the dynamic variations of ISI, rate distribution of ISI, and changes in adjacent ISI in our CSNN when exposed to white Gaussian stimulus. Three eigenvectors are extracted from three patterns under white Gaussian stimulus: the highest ISI is extracted from the ISI time domain diagram, the highest percentage of ISI is extracted from the ISI histogram, the highest disparity between neighboring ISI is extracted from the joint ISI distribution.

2. Coding pattern of CSNN under impulse stimulus

**Figure 3:**
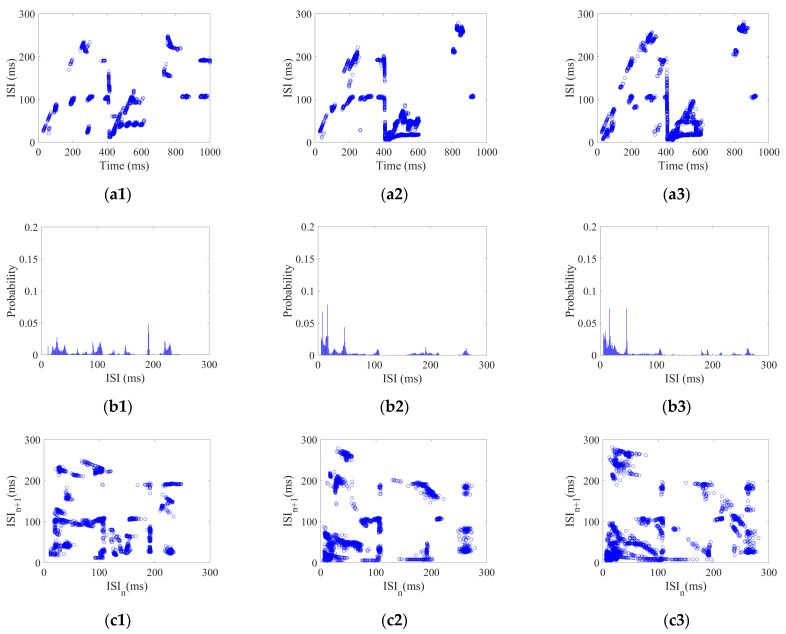
ISI coding pattern of the CSNN under impulse stimulus: (**a1**–**a3**) ISI time domain diagrams; (**b1**–**b3**) ISI histograms; (**c1**–**c3**) joint ISI distributions.

Each neuron in the CSNN received impulse stimulus with a strength range of 5–25 mA with 2.5 mA/step. Using the impulse stimulus strength $A_{s}$ of 5, 15, and 25 as examples, their three aspects of ISI coding are shown in Figure 3.

Figure 3(a1–a3) show ISI time domain diagrams under $A_{s}$ of 5, 15, and 25 mA; Figure 3(b1–b3) show ISI histograms under $A_{s}$ of 5, 15, and 25 mA; Figure 3(c1–c3) show joint ISI distributions under $A_{s}$ of 5, 15, and 25 mA. The neural coding pattern illustrated in Figure 3 reflects the dynamic variations of ISI, rate distribution of ISI, and changes in adjacent ISI in our CSNN when exposed to impulse stimulus. Three eigenvectors are extracted from three patterns under impulse stimulus: the highest ISI is extracted from the ISI time domain diagram; the highest percentage of ISI is extracted from the ISI histogram; and the highest disparity between neighboring ISI is extracted from the joint ISI distribution.

3. Coding pattern of CSNN under AC magnetic field stimulus

**Figure 4:**
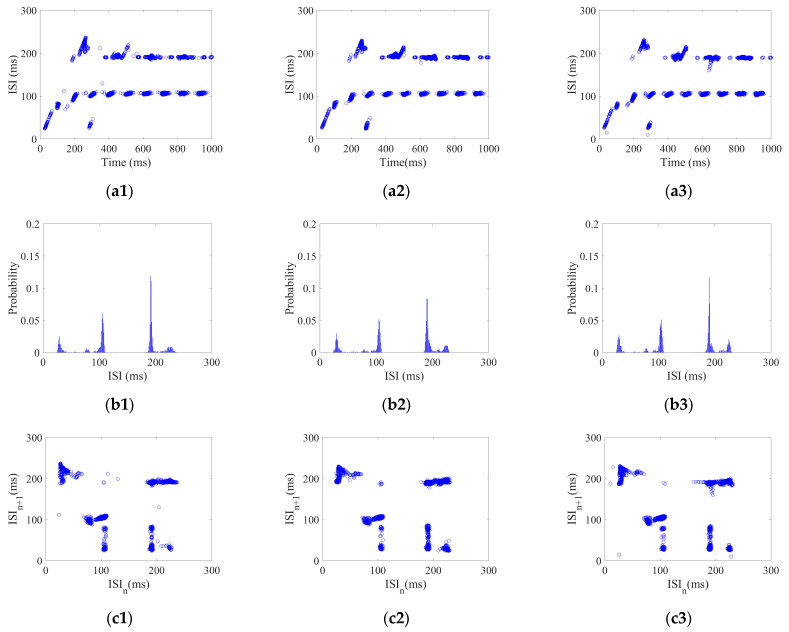
ISI coding pattern of the CSNN under AC magnetic field stimulus. (**a1**–**a3**) ISI time domain diagrams; (**b1**–**b3**) ISI histograms; (**c1**–**c3**) joint ISI distributions.

Each neuron in the CSNN received AC magnetic field stimulus with a strength range of 5–25 mV with 2.5 mV/step. Using the AC magnetic field stimulus amplitude $A_{c}$ of 5, 15, and 25 mV as examples, their three aspects of ISI coding are shown in Figure 4.

Figure 4(a1–a3) show ISI time domain diagrams under $A_{c}$ of 5, 15, and 25 mV; Figure 4(b1–b3) show ISI histograms under $A_{c}$ of 5, 15, and 25 mV; Figure 4(c1–c3) show joint ISI distributions under $A_{c}$ of 5, 15, and 25 mV. The neural coding pattern illustrated in Figure 4 reflects the dynamic variations of ISI, rate distribution of ISI, and changes in adjacent ISI in our CSNN when exposed to AC magnetic field stimulus. Three eigenvectors are extracted from three patterns under AC magnetic field stimulus: the highest ISI is extracted from the ISI time domain diagram, the highest percentage of ISI is extracted from the ISI histogram, the highest disparity between neighboring ISI is extracted from the joint ISI distribution.

### 2.3. The SNC of CSNN

Based on the time coding of CSNN, we employ a cosine similarity algorithm to explore similarity within the classes, while employing K-means clustering algorithm to explore the disparity between classes. In this subsection, we introduce these two algorithms and provide details on their parameters.

#### 2.3.1. Cosine Similarity Algorithm

The cosine similarity algorithm is chosen because it effectively captures the similarity between eigenvectors, ensuring that the structural resemblance of ISI coding patterns is accurately measured. This method is widely used for measuring the similarity between eigenvectors. This method is as follows [41]:

(15) $$cos\theta =\frac{V_{1}\cdot V_{2}}{\left|V_{1}\right|\left|V_{2}\right|}$$

where $V_{1}$ and $V_{2}$ denote two various samples containing the chosen eigenvector. In this study, we set a similarity threshold of 0.8 based on empirical testing. If the cosine similarity between two eigenvectors exceeds this threshold, we consider them to belong to the same class. This threshold ensures that only highly similar responses are clustered together, which helps avoid misclassification in the analysis.

#### 2.3.2. K-Means Clustering Algorithm

K-means clustering algorithm is well-suited for this task, efficiently grouping coding patterns into clusters and enabling the analysis of coding differences across stimuli, which helps to identify different responses across varying stimuli and allows for a clear analysis of coding differences. K-means clustering algorithm was utilized to calculate the time coding disparity of CSNN under various stimuli. The steps of the algorithm are the following [42]:

- Decide the number of clusters K: We set K = 3 because we aim to categorize the data into three distinct clusters corresponding to the different time coding patterns of CSNN.
- Decide the number of eigenvectors H: We set H = 3 because one sample has three eigenvectors. Therefore, a sample with three eigenvectors is randomly chosen as the initial cluster center for each cluster.
- Assign samples to clusters: For every sample, the Euclidean distance between the sample and each cluster center is computed; then, each sample is assigned to the cluster closest to its center.
- Recompute the cluster centers: Compute the new cluster center by averaging each eigenvector across all samples.
- Repeat until convergence: Repeat these steps until the classification outcomes for each sample remain unchanged.

## 3. Results

This section first introduces the experimental settings. Then, we compute the CC and SPL of CSNN. Finally, we then investigate the SNC of CSNN based on time coding under various exterior stimuli.

### 3.1. Experimental Settings

In this subsection, the experimental settings are introduced, including the parameters of the Izhikevich neuron model and the synaptic plasticity model.

#### 3.1.1. Parameters of Izhikevich Neuron Model

Following [43], the proportion of excitatory to inhibitory Izhikevich neuron models is 4:1. Hence, we stochastically distribute them with same ratio of 4:1 within the SNNs. Following [44], Table 2 presents the dimensionless parameters and corresponding values for a, b, c, and d in the Izhikevich neuron models utilized, encompassing excitatory and inhibitory. These parameters are crucial for modeling the firing behavior of neurons. Thus, the parameters in Table 2 are the key components of the Izhikevich neuron model employed in this study.

#### 3.1.2. Parameters of the Synaptic Plasticity Model

Following [33,34], Table 3 shows the parameters and corresponding values used in synaptic plasticity model. These parameters help simulate changes in synaptic weights in response to neuronal activity.

Biological research indicates that the bio-STD is stochastically distributed in interval [0.1, 40] ms [16]. We therefore incorporate an STD into the synaptic plasticity model.

### 3.2. Results of Topological Characteristics of CSNN

To evaluate the topological characteristics of CSNN, we computed the ${\tilde{C}}_{w}$ and ${\tilde{L}}_{w}$ of CSNN as shown in Table 4.

Table 4 presents the ${\tilde{C}}_{w}$ and ${\tilde{L}}_{w}$ of CSNN. ${\tilde{C}}_{w}=0.49$ falls within the range of ${\tilde{C}}_{w}$ of human brain (${\tilde{C}}_{w}<$ 0.88) [21]; ${\tilde{L}}_{w}=3.37$ falls within the range of ${\tilde{L}}_{w}$ of human brain (1.7 $<{\tilde{L}}_{w}<$ 5.1) [21]. These results indicate that CSNN is a bio-rational brain-inspired model and effectively balances local and global connectivity.

### 3.3. Time Coding of CSNN

In this subsection, we investigated the similarity within classes and disparity between classes of CSNN.

#### 3.3.1. The Time Coding Similarity Under the Same Stimulus

The cosine similarity algorithm serves to assess the similarity among time coding under various strengths for each stimulus. For white Gaussian stimulus (5–25 dBW, 2.5 dBW/step), impulse stimulus (5–25 mA, 2.5 mA/step), and AC magnetic field stimulus (5–25 mV, 2.5 mV/step), each strength value corresponds to a unique time coding, which we considered as one sample. Hence, nine samples were collected for each stimulus. For every sample, we extracted the highest ISI (eigenvector 1), the highest percentage of ISI (eigenvector 2), and the highest disparity between neighboring ISI (eigenvector 3). Each sample’s three eigenvectors together form a three-dimensional eigenvector, which serves as the vector $V_{1}$ (or $V_{2}$) in Equation (13).

For each type of stimulus, the cosine similarity between samples under any two stimuli of different strengths is analyzed and the mean value is calculated. This method ensures that the cosine similarity calculation can fully reflect the overall features of the time code, rather than just the similarities of individual features. These means are presented in Table 5.

Table 5 illustrates that cosine similarity of CSNN under three stimuli was near to one, which suggests that the CSNN demonstrates significant time coding similarity when subjected to varying strengths of the same stimulus.

Figure 2, Figure 3 and Figure 4 illustrate the ISI coding patterns of CSNN under different stimuli. The cosine similarity values in Table 5 further support this observation: under the same stimulus condition, the CSNN exhibits highly similar SNC patterns (cosine similarity > 0.99), indicating that SNC is characterized by similarity within the classes.

#### 3.3.2. Time Coding Disparity Under Various Stimuli

We applied the K-means clustering algorithm to analyze the differences in time coding under various stimuli. These simulation parameters align with those for assessing the previously discussed similarities. We computed means of the clustering accuracies of CSNN’s time coding under various stimuli. The classification accuracies can be found in Table 6.

The classification accuracy for ISI coding patterns utilizing Eigenvectors 1 + Eigenvectors 2 + Eigenvectors 3 is highest as shown in Table 6, which indicates that the ISI coding patterns of CSNN have the largest disparity under various stimuli.

Figure 2, Figure 3 and Figure 4 illustrate ISI coding patterns of CSNN under different stimuli. The classification accuracies in Table 6 further support this observation: different stimuli lead to distinct coding patterns, as evidenced by the varying classification performances. Specifically, individual eigenvectors achieve moderate accuracy (around 72–76%), whereas the combination of all three eigenvectors significantly enhances classification performance (97.32%). This suggests that SNC patterns exhibit notable differences across stimuli.

Overall, our findings indicate that the CSNN exhibits a marked SNC characterized by similarity within the classes and disparity between classes.

## 4. Discussion

In this section, we first elucidate the mechanism of SNC. Then, we discuss the comparison with other research and limitations.

### 4.1. The Mechanism of SNC

To elucidate mechanism of SNC, we explore the relationship between the SNC and the synaptic plasticity.

#### 4.1.1. The Synaptic Weight

**Figure 5:**
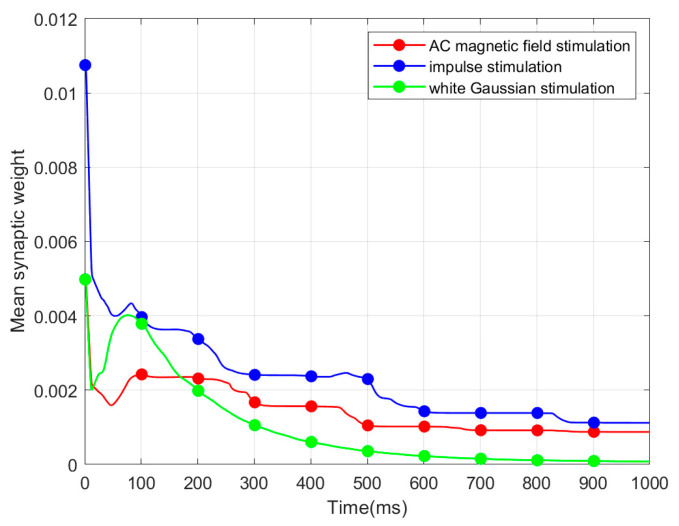
Evolution of MSW.

SNN is connected via synaptic plasticity in accordance with topological connection. Consequently, we investigated the synaptic weight. The mean synaptic weight (MSW) refers to mean weight of all the synapses within an SNN. Based on the same strength of three stimuli mentioned above, we illustrate the evolution of MSWs of the CSNN under various stimuli, as presented in Figure 5.

Based on Figure 5, three evolution processes of MSW exhibit a comparable tendency. MSW shows a considerable decrease in the initial 300 ms, followed by a stabilization period from 300 ms to 1000 ms.

In our study, the MSW decreases in the initial 300 ms. This behavior occurs due to the continuous exterior stimuli applied from the start of the simulation. Once the network has fully adapted to the external input, the MSW stabilizes. The three exterior stimuli exert a consistent influence on the CSNN throughout the simulation.

#### 4.1.2. Relevance Analysis

**Figure 6:**
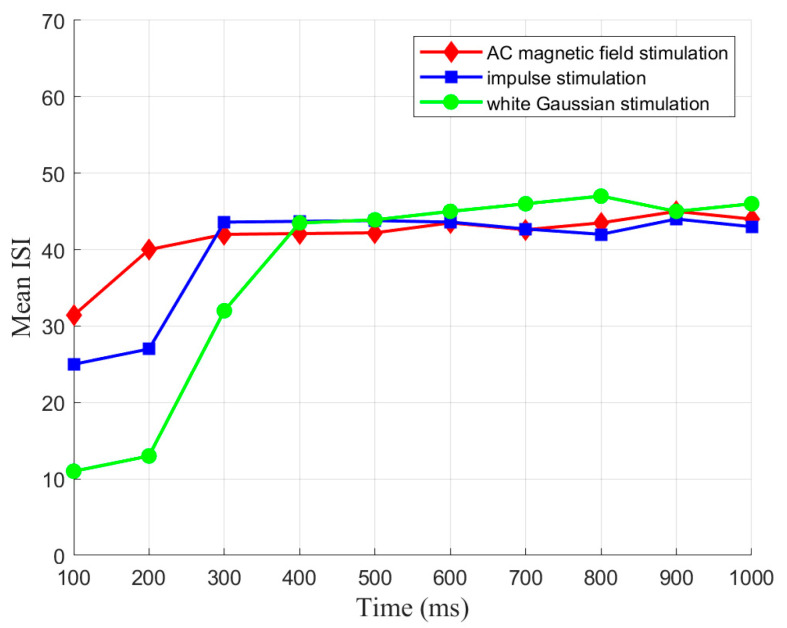
Evolution of mean ISI.

The CSNN generates the relevant time coding under a specific stimulus, which forms SNC. To elucidate the mechanism of SNC, we established the relationship between MSW and mean ISI. The mean ISI represents the mean of all neuronal ISI within an SNN over a given period. Based on the same strength of three stimuli mentioned earlier, we initially show the evolution of mean ISI of the CSNN under various stimuli over a 100 ms interval, as depicted in Figure 6.

Based on Figure 6, these evolution processes of mean ISI exhibit a comparable tendency. Mean ISI shows a considerable increase in the initial 300 ms, followed by a stabilization period from 300 ms to 1000 ms. The continuous exterior stimuli throughout the simulation also drive the observed increase and stabilization of mean ISI. Initially, the network adapts to the exterior stimuli, increasing mean ISI. Once the network reaches equilibrium, the mean ISI stabilizes.

Subsequently, we established the relationship between SNC and synaptic plasticity through the Pearson correlation, which is expressed as follows:

(16) $$R=\frac{\sum_{i=1}^{n}\left(X_{i}-\bar{X}\right)\left(Y_{i}-\bar{Y}\right)}{\sqrt{\sum_{i=1}^{n}{\left(X_{i}-\bar{X}\right)}^{2}}\sqrt{\sum_{i=1}^{n}{\left(Y_{i}-\bar{Y}\right)}^{2}}}$$

where X and Y represent the samples; n denotes the size of the sample. In order to assess the significance of R, the t-test is utilized, which is described as follows:

(17) $$t_{test}=R/\sqrt{1-R^{2}/\left(n-2\right)}$$

The 0.01 level of significance is indicated by “**” and the 0.05 level of significance is indicated by “*”.

In this research, X represents MSW over a 100 ms time interval; Y represents mean ISI for the same duration; n is set to 10. The R between MSW and mean ISI in the CSNN under three stimuli with various strengths are presented in Table 7.

Table 7 indicates a significant correlation at the 0.01 level between the three mean ISIs and their respective MSWs across three different stimuli, which suggests that inherent factor of SNC is synaptic plasticity.

Table 4 presents the CC and SPL of CSNN. These structural metrics reflect the organization of synaptic connectivity and support the observed SNC formation. Additionally, Table 5 demonstrates the cosine similarity of the ISI coding patterns under different stimuli strengths. The high similarity values indicate that the SNC maintains stability despite variations in exterior stimulus intensity. Furthermore, the classification accuracy results in Table 6 highlight the discriminative power of SNC across different stimulus conditions. By integrating Figure 2, Figure 3, Figure 4, Figure 5 and Figure 6 and Table 4, Table 5, Table 6 and Table 7, we establish a comprehensive connection between the ISI, synaptic plasticity, and network topology. The results demonstrate that the adaptation of synaptic weights directly impacts the coding pattern, reinforcing the role of synaptic plasticity in SNC formation and stability.

### 4.2. Comprehensive Discussion

In this section, we discuss the comparison with other methods and limitations of CSNN.

#### 4.2.1. Comparison with Other Methods

Our findings significantly contribute to the brain-inspired models, especially regarding neural coding. The performance of SNC is extremely dependent on brain-inspired models. Thus, we propose a bio-rational brain-inspired model. In terms of topology, our CSNN model is inspired by the topological characteristics of bio-FBN, in contrast to previous studies [24,25], which investigated topologies exhibiting SF or SW properties. In terms of synaptic plasticity model, our CSNN has a bio-STD and co-regulate of ES and IS, in contrast to the previous study [15], which focused on fixed STD. In terms of neural coding, our CSNN represents has significant SNC, in contrast to the previous study [45], which focused on the SNC of SFSNN. These comparisons illustrate the superiority of our method.

#### 4.2.2. Limitations

While this study provides insights into SNC of CSNN, several limitations should be acknowledged. First, although the topology of the CSNN aligns with bio-rationality, it does not fully capture the true connectivity patterns observed in biological neural networks. Furthermore, real-world implementations often encounter continuously changing and time-varying stimuli. However, this study does not investigate how CSNNs respond to such dynamic inputs. Finally, while a strong correlation between MSW and ISI is established, the study does not provide a causal explanation. A more detailed mechanistic analysis is necessary to confirm how synaptic plasticity directly affects SNC.

## 5. Conclusions

This study proposed CSNN, a brain-inspired model characterized by bio-rationality. Its topology combines the SW property and SF property. To investigate the SNC of CSNN under various strengths of various stimuli, we used a K-means clustering algorithm and a cosine similarity algorithm. To elucidate its mechanism, we discuss the relationship between SNC and synaptic plasticity.

The following main conclusions are drawn from this work: (1) The CSNN exhibits marked time coding similarity under various strengths of the same stimulus; the CSNN exhibits marked SNC under various stimuli. (2) Our discussion indicates that the inherent factor of the SNC is synaptic plasticity. Investigating SNC and its underlying mechanisms can enhance our comprehension of intricate brain cognitive functions and the processes involved in information handling, while also fostering advancements in artificial intelligence.

For our future work, we aim to construct a more bio-rationality brain-inspired model, and will apply this brain-inspired model based on SNC to other pattern recognition tasks such as image recognition and speech recognition. Second, investigating how CSNN respond to time-varying exterior stimuli remains an essential avenue for future work, which could provide insights into their robustness and efficiency in processing real-world signals. Finally, while the present study identifies a significant correlation between MSW and ISI, future work should focus on uncovering the causal mechanisms underlying this relationship.

## Figures and Tables

**Table 1:** γ and σ of the complex network with different $P_{n}$.

$P_{n}$	0.1	0.2	0.3	0.4	0.5	0.6	0.7	0.8	0.9	1.0
γ	1.49	1.83	2.15	2.38	2.51	2.76	2.82	2.84	3.01	3.12
σ	1.33	1.50	1.89	1.72	1.69	1.67	1.61	1.50	1.48	1.12

**Table 2:** Parameters of the Izhikevich neuron models.

Parameter	Instruction	Value
a	Timescale for u	Excitement: 0.02
		Inhibition: 0.02
b	Sensitivity of u to sub-threshold fluctuations in v	Excitement: 0.20
		Inhibition: 0.25
c	Reset value of v	Excitement: −65
		Inhibition: −65
d	Reset value of u	Excitement: 8
		Inhibition: 2

**Table 3:** Parameters of the synaptic plasticity model.

Parameter	Description	Value
E [33]	Reversible synaptic potential	Excitement: 0 mV
		Inhibition: −70 mV
α [33]	Forward rate constant for neurotransmitters	Excitement: 2
		Inhibition: 0.9
β [33]	Reverse rate constant for neurotransmitters	Excitement: 1
		Inhibition: 0.1
$\mu_{ex}$ [34]	Decay constant of the ES weight	3 ms
$\mu_{in}$ [34]	Decay constant of the IS weight	5 ms
$g_{max}$ [34]	Maximum value of the synaptic weight	0.015
$A_{+}$ [34]	Maximum correction value when the ES weight is increased	0.1
$A_{-}$ [34]	Minimum correction value when the ES weight is reduced	0.105
$B_{+}$ [34]	Maximum correction value when the IS weight is increased	0.02
$B_{-}$ [34]	Minimum correction value when the IS weight is reduced	0.03
$t_{+}$ [34]	Interval for the presynaptic and postsynaptic firing of neurons when the synaptic weights are increased	20 ms
$t_{-}$ [34]	Interval for the presynaptic and postsynaptic firing of neurons when the synaptic weights are reduced	20 ms

**Table 4:** The ${\tilde{C}}_{w}$ and ${\tilde{L}}_{w}$ of CSNN.

Topological Characteristics	Results
${\tilde{C}}_{w}$	0.49
${\tilde{L}}_{w}$	3.37

**Table 5:** Cosine similarity.

Stimuli	Cosine Similarity
White Gaussian stimulus	0.9970
Impulse stimulus	0.9982
AC magnetic field stimulus	0.9957

**Table 6:** The classification accuracies.

Eigenvectors	Classification Accuracy
Eigenvectors 1	72.54%
Eigenvectors 2	76.33%
Eigenvectors 3	72.16%
Eigenvectors 1 + Eigenvectors 2 + Eigenvectors 3	97.32%

**Table 7:** The R for various stimuli.

	White Gaussian Stimulus (dBW)	Impulse Stimulus (mA)	AC Magnetic Field Stimulus (mV)
Mean ISI (under 5.00)	−0.940 **	−0.910 **	−0.963 **
Mean ISI (under 10.00)	−0.934 **	−0.882 **	−0.953 **
Mean ISI (under 15.00)	−0.912 **	−0.891 **	−0.969 **
Mean ISI (under 20.00)	−0.943 **	−0.907 **	−0.945 **
Mean ISI (under 25.00)	−0.927 **	−0.881 **	−0.978 **

The 0.01 level of significance is indicated by “**”.

## Data Availability

The original contributions presented in this study are included in the article. Further inquiries can be directed to the corresponding author(s).

## References

[1] Norman-Haignere S.V., Feather J., Boebinger D., Brunner P., Ritaccio A., McDermott J.H., Schalk G., Kanwisher N. (2022). A Neural Population Selective for Song in Human Auditory Cortex. Curr. Biol..

[2] Panzeri S., Harvey C.D., Piasini E., Latham P.E., Fellin T. (2017). Cracking the Neural Code for Sensory Perception by Combining Statistics, Intervention, and Behavior. Neuron.

[3] Baez-Santiago M.A., Reid E.E., Moran A., Maier J.X., Marrero-Garcia Y., Katz D.B. (2016). Dynamic taste responses of parabrachial pontine neurons in awake rats. J. Neurophysiol..

[4] Tang F., Zhang J., Zhang C., Liu L. (2024). Brain-Inspired Architecture for Spiking Neural Networks. Biomimetics.

[5] Passias A., Tsakalos K.A., Kansizoglou I., Kanavaki A.M., Gkrekidis A., Menychtas D., Aggelousis N., Michalopoulou M., Gasteratos A., Sirakoulis G.C. (2024). A Biologically Inspired Movement Recognition System with Spiking Neural Networks for Ambient Assisted Living Applications. Biomimetics.

[6] Yu D., Wang G., Li T., Ding Q., Jia Y. (2023). Filtering Properties of Hodgkin–Huxley Neuron on Different Time-Scale Signals. Commun. Nonlinear Sci. Numer. Simul..

[7] Kamal N., Singh J. (2021). A Highly Scalable Junctionless FET Leaky Integrate-and-Fire Neuron for Spiking Neural Networks. IEEE Trans. Electron Devices.

[8] Elkaranshawy H.A., Aboukelila N.M., Elabsy H.M. (2021). Suppressing the Spiking of a Synchronized Array of Izhikevich Neurons. Nonlinear Dyn..

[9] Wang G., Yang L., Zhan X., Li A., Jia Y. (2022). Chaotic Resonance in Izhikevich Neural Network Motifs under Electromagnetic Induction. Nonlinear Dyn..

[10] Liu D., Guo L., Wu Y., Lv H., Xu G. (2021). Antiinterference Function of Scale-Free Spiking Neural Network under AC Magnetic Field Stimulation. IEEE Trans. Magn..

[11] She X., Long Y., Kim D., Mukhopadhyay S. (2021). ScieNet: Deep Learning with Spike-Assisted Contextual Information Extraction. Pattern Recognit..

[12] He H., Shen W., Zheng L., Guo X., Cline H.T. (2018). Excitatory Synaptic Dysfunction Cell-Autonomously Decreases Inhibitory Inputs and Disrupts Structural and Functional Plasticity. Nat. Commun..

[13] Scekic-Zahirovic J., Sanjuan-Ruiz I., Kan V., Megat S., De Rossi P., Dieterlé S., Cassel R., Jamet M., Kessler P., Wiesner D. (2021). Cytoplasmic FUS Triggers Early Behavioral Alterations Linked to Cortical Neuronal Hyperactivity and Inhibitory Synaptic Defects. Nat. Commun..

[14] Xue M., Atallah B.V., Scanziani M. (2014). Equalizing Excitation–Inhibition Ratios across Visual Cortical Neurons. Nature.

[15] Zhao D., Zeng Y., Li Y. (2022). BackEISNN: A Deep Spiking Neural Network with Adaptive Self-Feedback and Balanced Excitatory–Inhibitory Neurons. Neural Netw..

[16] Taherkhani A., Belatreche A., Li Y., Cosma G., Maguire L.P., McGinnity T.M. (2020). A Review of Learning in Biologically Plausible Spiking Neural Networks. Neural Netw..

[17] Zhang M., Wu J., Belatreche A., Pan Z., Xie X., Chua Y., Li G., Qu H., Li H. (2020). Supervised Learning in Spiking Neural Networks with Synaptic Delay-Weight Plasticity. Neurocomputing.

[18] Barthelemy M. (2018). Morphogenesis of Spatial Networks.

[19] Nemzer L.R., Cravens G.D., Worth R.M., Motta F., Placzek A., Castro V., Lou J.Q. (2021). Critical and ictal phases in simulated EEG signals on a small-world network.Front. Comput. Neurosci..

[20] Keerthana G., Anandan P., Nandhagopal N. (2021). Enhancing the robustness and security against various attacks in a scale: Free network. Wirel. Pers. Commun..

[21] Van Den Heuvel M.P., Stam C.J., Boersma M., Hulshoff Pol H.E. (2008). Small-World and Scale-Free Organization of Voxel-Based Resting-State Functional Connectivity in the Human Brain. NeuroImage.

[22] Liu Y., Liang M., Zhou Y., He Y., Hao Y., Song M., Yu C., Liu H., Liu Z., Jiang T. (2008). Disrupted Small-World Networks in Schizophrenia. Brain.

[23] Stylianou O., Kaposzta Z., Czoch A., Stefanovski L., Yabluchanskiy A., Racz F.S., Ritter P., Eke A., Mukli P. (2022). Scale-Free Functional Brain Networks Exhibit Increased Connectivity, Are More Integrated and Less Segregated in Patients with Parkinson’s Disease Following Dopaminergic Treatment. Fractal Fract..

[24] Tsakalos K.-A., Sirakoulis G.C., Adamatzky A., Smith J. (2022). Protein Structured Reservoir Computing for Spike-Based Pattern Recognition. IEEE Trans. Parallel Distrib. Syst..

[25] Reis A.S., Brugnago E.L., Caldas I.L., Batista A.M., Iarosz K.C., Ferrari F.A.S., Viana R.L. (2021). Suppression of Chaotic Bursting Synchronization in Clustered Scale-Free Networks by an External Feedback Signal. Chaos Interdiscip. J. Nonlinear Sci..

[26] Guo L., Song Y., Wu Y., Xu G. (2023). Anti-Interference of a Small-World Spiking Neural Network against Pulse Noise. Appl. Intell..

[27] Steinmetz N.A., Zatka-Haas P., Carandini M., Harris K.D. (2019). Distributed Coding of Choice, Action and Engagement across the Mouse Brain. Nature.

[28] Callier T., Suresh A.K., Bensmaia S.J. (2019). Neural Coding of Contact Events in Somatosensory Cortex. Cereb. Cortex.

[29] Zhu Z., Wang R., Zhu F. (2018). The Energy Coding of a Structural Neural Network Based on the Hodgkin–Huxley Model. Front. Neurosci..

[30] Du L., Cao Z., Lei Y., Deng Z. (2019). Electrical Activities of Neural Systems Exposed to Sinusoidal Induced Electric Field with Random Phase. Sci. China Technol. Sci..

[31] Staszko S.M., Boughter J.D., Fletcher M.L. (2022). The Impact of Familiarity on Cortical Taste Coding. Curr. Biol..

[32] Izhikevich E.M. (2003). Simple Model of Spiking Neurons. IEEE Trans. Neural Netw..

[33] Destexhe A., Mainen Z.F., Sejnowski T.J. (1994). An Efficient Method for Computing Synaptic Conductances Based on a Kinetic Model of Receptor Binding. Neural Comput..

[34] Kleberg F.I., Fukai T., Gilson M. (2014). Excitatory and Inhibitory STDP Jointly Tune Feedforward Neural Circuits to Selectively Propagate Correlated Spiking Activity. Front. Comput. Neurosci..

[35] Dan W., Xiao-Zheng J. (2012). On Weighted Scale-Free Network Model with Tunable Clustering and Congestion. Acta Phys. Sin..

[36] Hartmann B., Sugár V. (2021). Searching for Small-World and Scale-Free Behaviour in Long-Term Historical Data of a Real-World Power Grid. Sci. Rep..

[37] Eguíluz V.M., Chialvo D.R., Cecchi G.A., Baliki M., Apkarian A.V. (2005). Scale-Free Brain Functional Networks. Phys. Rev. Lett..

[38] Piersa J., Piekniewski F., Schreiber T. (2010). Theoretical Model for Mesoscopic-Level Scale-Free Self-Organization of Functional Brain Networks. IEEE Trans. Neural Netw..

[39] Li G., Luo Y., Zhang Z., Xu Y., Jiao W., Jiang Y., Huang S., Wang C. (2019). Effects of Mental Fatigue on Small-World Brain Functional Network Organization. Neural Plast..

[40] Diykh M., Li Y., Wen P. (2017). Classify epileptic EEG signals using weighted complex networks based community structure detection. Expert Syst. Appl..

[41] Liu Y., Zeng Y., Li R., Zhu X., Zhang Y., Li W., Li T., Zhu D., Hu G. (2024). A Random Particle Swarm Optimization Based on Cosine Similarity for Global Optimization and Classification Problems. Biomimetics.

[42] Liu G., Li M., Wang H., Lin S., Xu J., Li R., Tang M., Li C. (2022). D3K: The Dissimilarity-Density-Dynamic Radius K-Means Clustering Algorithm for scRNA-Seq Data. Front. Genet..

[43] Vogels T.P., Sprekeler H., Zenke F., Clopath C., Gerstner W. (2011). Inhibitory Plasticity Balances Excitation and Inhibition in Sensory Pathways and Memory Networks. Science.

[44] Izhikevich E.M. (2004). Which Model to Use for Cortical Spiking Neurons?. IEEE Trans. Neural Netw..

[45] Guo L., Hou L., Wu Y., Lv H., Yu H. (2020). Encoding specificity of scale-free spiking neural network under different external stimulations. Neurocomputing.

